# The Strengths and Difficulties Questionnaire Predicts Concurrent Mental Health Difficulties in a Transdiagnostic Sample of Struggling Learners

**DOI:** 10.3389/fpsyg.2020.587821

**Published:** 2020-11-12

**Authors:** Annie Bryant, Jacalyn Guy, The CALM Team, Duncan Astle, Joni Holmes

**Affiliations:** ^1^Department of Clinical Psychology, Faculty of Medicine and Health Sciences, University of East Anglia, Norwich, United Kingdom; ^2^Medical Research Council (MRC) Cognition and Brain Sciences Unit, University of Cambridge, Cambridge, United Kingdom

**Keywords:** Strength and Difficulties Questionnaire, mental health, learning difficulties, screening, transdiagnostic

## Abstract

Children and adolescents with developmental problems are at increased risk of experiencing mental health problems. The Strengths and Difficulties Questionnaire (SDQ) is widely used as a screener for detecting mental health difficulties in these populations, but its use thus far has been restricted to groups of children with diagnosed disorders (e.g., ADHD). Transdiagnostic approaches, which focus on symptoms and soften or remove the boundaries between traditional categorical disorders, are increasingly adopted in research and practice. The aim of this study was to assess the potential of the SDQ to detect concurrent mental health problems in a transdiagnostic sample of children. The sample were referred by health and educational professionals for difficulties related to learning (*N* = 389). Some had one diagnosis, others had multiple, but many had no diagnoses. Parent-rated SDQ scores were significantly positively correlated with parent ratings of mental health difficulties on the Revised Child Anxiety and Depression Scale (RCADS). Ratings on the SDQ Emotion subscale significantly predicted the likelihood of having concurrent clinical anxiety and depression scores. Ratings on the Hyperactivity subscale predicted concurrent anxiety levels. These findings suggest the SDQ could be a valuable screening tool for identifying existing mental health difficulties in children recognized as struggling, as it can be in typically developing children and those with specific diagnoses.

## Introduction

Mental health problems affect up to 15% of the school-age population and are more common in children with learning-related problems than typically developing children (Emerson and Hatton, 2007; Francis et al., 2019; Public Health England, 2019). Yet less than a third of children with learning problems receive mental health support (Dekker and Koot, 2003; Strømme and Diseth, 2007). This leads to poor long-term outcomes for individuals and increased economic and societal costs due to elevated mental health care needs, greater risks of school-exclusion and unemployment, and increased levels of antisocial behavior (Green et al., 2005; Joint Commissioning Panel for Mental Health, 2013; Ford et al., 2018; Anderson et al., 2019). The Strengths and Difficulties Questionnaire (SDQ; Goodman, 1997), a brief behavioral questionnaire that can be completed by teachers, parents, or children, has been used widely in community samples to predict psychopathology (Goodman et al., 2003; Goodman and Goodman, 2009). The aim of this study is to investigate whether parent ratings on the SDQ can be used to predict concurrent mental health symptoms in children with learning-related problems who are likely to be at increased risk of anxiety and depression. The sample are a large heterogeneous cohort of struggling learners recruited to the Centre for Attention, Learning and Memory (CALM) study. It includes children with learning-related difficulties such as dyslexia and ADHD, and others with impairments in attention, learning or memory that did not meet diagnostic thresholds but nonetheless were recognized by a health or educational professional as compromising the child’s school progress.

Children with developmental difficulties are at increased risk of mental health problems. Internalizing affective disorders, like anxiety and depression, are more common in children with diagnosed developmental disorders such as attention deficit hyperactivity disorder (ADHD; Biederman et al., 1996; Ford et al., 2003; Larson et al., 2011) and autism spectrum disorder (ASD; de Bruin et al., 2007; Simonoff et al., 2008; White et al., 2009; Strang et al., 2012; Rodgers and Ofield, 2018). Learning-related difficulties are also associated with heightened levels of affective symptoms. Children with specific language impairment (SLI, now termed developmental language disorder, DLD) are at increased risk of emotional problems and depressive disorders (Goh et al., 2013), and a diagnosis of DLD in early childhood significantly increases the risk of anxiety disorders in adolescence and young adulthood (Beitchman et al., 2001). Elevated symptoms of anxiety (Murray, 1978) and depression (Boetsch et al., 1996) are also common in children with dyslexia and literacy difficulties (Carroll et al., 2005). Children with comorbid developmental or learning problems and mental ill-health typically have lower self-esteem (Stevenson and Romney, 1984), poorer relationships with family and peers, and elevated levels of aggression (Kim et al., 2000) compared to children with only one condition. Providing these children with timely support is therefore paramount and underscores the need to evaluate tools for identifying symptoms of mental health disorders in at-risk groups.

Traditional models of identifying mental ill-health involve referrals and assessments by clinicians. Demands on health services often outstrip resources (Belfer, 2008; Hunt and Eisenberg, 2010), meaning alternative approaches to identifying children at-risk of poor mental health have been evaluated. These include school-wide screening programmes, curriculum-based pupil education, staff training, and teacher, parent or child questionnaires (see Anderson et al., 2019 for a review). These methods have been linked with increased support and better long-term mental health outcomes relative to children who have been identified through healthcare settings (Ford et al., 2008; Nemeroff et al., 2008; Sayal et al., 2010; Husky et al., 2011; Lyon et al., 2016). Brief questionnaires measuring psychopathological symptoms are increasingly used to identify children who need to access mental health services. Typical measures include the brief Rutter questionnaires (Elander and Rutter, 1996), the more comprehensive Child Behavior Checklist (CBCL; Achenbach and Edelbrock, 1991), and the SDQ (Goodman, 1997). The SDQ asks about positive and negative attributes across 5 scales with a total of 25 items: Emotional symptoms, Conduct problems, Hyperactivity/Inattention, Peer Relationship Problems and Prosocial Behavior. One advantage of the SDQ is that each scale is comprised of only five items. It is also widely and freely available for use by teachers and parents. Other tools such as the CBCL or direct assessments of children’s mental health symptoms (e.g., the Revised Child and Anxiety and Depression Scale (RCADS; Chorpita et al., 2000) are more commonly used in clinical settings. Further, the SDQ correlates strongly with the longer Rutter and CBCL scales and has been used successfully to predict the likelihood of psychiatric illness in the Mental Health of Children and Young People in England Survey (MHCYP) in 1999 (Meltzer et al., 2003), 2004 (Green et al., 2005) and 2017 (Sadler et al., 2018). These data show SDQ ratings are linearly related to the prevalence of a psychopathological disorders in community samples (Goodman and Goodman, 2009).

In terms of at-risk groups, SDQ scores provide a good indication of comorbid affective problems in children with ADHD, with stronger relationships for externalizing than internalizing problems (Bekker et al., 2016). In adolescents with ASD, SDQ scores at age 12 predict severe mood problems at age 16 (Simonoff et al., 2012), and in children with dyslexia, higher SDQ scores are associated with lower self-esteem (Terras et al., 2009). The SDQ has been used with groups of children with intellectual disabilities, but its utility with severely impaired learners is limited (Emerson et al., 2010). To date, there have been no investigations into the utility of the SDQ for identifying symptoms of mental health problems in the common struggling learner.

In the present study, we assessed the potential utility of the SDQ for mental health screening in a large sample of children experiencing learning-related problems. Children with developmental problems are typically categorized into discrete and highly selective diagnostic groups such as ADHD, ASD, or specific learning and communication disorders according to symptom criteria outlined in international classification systems such as the Diagnostic and Statistical Manual-5 (DSM-5; American Psychiatric Association, 2013). These criteria constitute a framework for defining children’s difficulties that is used both to guide practical support in schools and clinics, to study the causes and characteristics of individuals who are struggling, and to frame our understanding of the links between developmental disorders and mental health problems. The problem with this traditional diagnostic nosology is that it fails to capture the variability, high levels of co-occurrence, and symptom overlap of common cognitive, behavioral and learning problems (Kotov et al., 2017). Contemporary thinking within developmental psychology is that we should adopt a transdiagnostic approach that softens or removes the boundaries between discrete categories of disorder (e.g., Coghill and Sonuga-Barke, 2012; Holmes et al., 2019).

In line with a transdiagnostic approach, we recruited a highly heterogeneous population of poor learners through the larger Centre for Attention, Learning and Memory (CALM) project (Holmes et al., 2019). The sample is representative of the majority of children who are struggling in the classroom. All children were referred by a health or educational professional for difficulties in attention, learning and/or memory. In this way, they were functionally defined as struggling. They did not fit traditional discrete categories of developmental and learning disorders; some had a single diagnosis, others had multiple diagnoses, but the majority were undiagnosed despite coming to the attention of a health or educational professional for experiencing difficulties that were affecting their school progress. Using this unique sample, the aim of this study is to provide a critical test of the utility of the parent-rated SDQ for identifying concurrent mental health problems in a transdiagnostic sample of struggling learners who are common in the classroom. This study was pre-registered on AsPredicted (#13396).

## Materials and Methods

### Procedure

Data were collected through the Centre for Attention, Learning and Memory (CALM) a developmental research clinic based at the MRC Cognition and Brain Sciences Unit, University of Cambridge, United Kingdom. Children aged 5–18 years were referred by health and education professionals for problems in attention, learning and/or memory. Families accepted into the study attended the clinic. Children completed a 3.5–4 h cognitive assessment, and parents/carers completed questionnaires measuring the child’s behavior, family history and mental health. See Holmes et al. (2019) for the study protocol. The study was approved by the local NHS Research Ethics Committee (13/EE/0157). Children assented to participation and parents/carers provided written informed consent on behalf of the child. For the majority of children, questionnaires were completed by parents, but the reporting agent in a small number of cases was a legal guardian/carer.

### Participants

The sample was drawn from the wider CALM project that includes 805 children who attended the CALM clinic between February 2014 and January 2019. The data analyzed include the SDQ (Goodman, 1997) and the RCADS-P (Chorpita et al., 2000). Children with complete data on these measures were included for analysis (*N* = 389). The RCADS-P was not introduced into the CALM testing protocol until December 2016, and it was only administered to children aged 8+. It was therefore only completed by 400 of the 805 children. This explains why the number of children included here does not correspond to the 805 in the full CALM cohort. Participant demographics, diagnoses, and referral routes are presented in Table 1.

**TABLE 1:** Participant characteristics including age, diagnosis, and referral route.

	**Mean (*SD*)**	**Min–Max**
**Age (years)**	9.73 (2.46)	5.42–18.58
	**N male**	**% male**
Gender	271	70

**Diagnosis**	**Total N**	**%**

None	207	53
ADHD	155	40
ASD	33	8
Anxiety	8	2
Depression	1	<1
**Learning related difficulties**		
Dyslexia	20	5
Dyspraxia	10	3
Dysgraphia	0	0
Dyscalculia	2	<1

**Referrer**	**Total N**	**%**

Education	223	57
Healthcare	157	40
SLT	9	2

*N = 389; ADHD, Attention Deficit Hyperactivity Disorder; ASD, Autism Spectrum Disorder, SLT, Speech and Language Therapist. Education referrers included special educational needs coordinators, specialist teachers, educational psychologists, private tutors and family workers. Healthcare referrers included clinical psychologists, child psychiatrists, pediatricians, and ADHD nurse practitioners. ADHD group includes children under assessment for ADHD. Percentages are rounded.*

### Materials

The SDQ queries positive and negative attributes displayed by the child in the past 6 months across five subscales: Emotional Symptoms (e.g., often unhappy, downhearted), Conduct Problems (e.g., fights with other children), Hyperactivity/Inattention (e.g., constantly fidgeting or squirming), Peer Relationship Problems (e.g., tends to play alone) and Prosocial Behavior (e.g., considerate of other people’s feelings). Each subscale includes five items and the parent/carer rates each item as either: Never = 0, Somewhat True = 1 or Certainly True = 2. SDQ total scores of 17 and above are considered to be abnormal. Raw scores are used in the analyses reported here. Clinical cut-off raw scores for the subscales (out of a possible 10) are: Emotion ≥ 5, Conduct ≥ 4, Hyperactivity ≥ 7, Peer Problems ≥ 4, Prosocial Behavior ≤ 4.

The RCADS-P is designed to assess children’s symptoms in relation to DSM anxiety and major depressive disorders (Chorpita et al., 2000). Parents rate the frequency of their child’s feelings and behaviors on 47 items (Never = 0, Sometimes = 1, Often = 2 or Always = 3). RCADS-P has five subscales corresponding to anxiety disorders: Separation Anxiety Disorder (e.g., fears being alone at home), Social Phobia (e.g., worries they might look foolish), Generalized Anxiety Disorder (e.g., worries bad things will happen to them), Panic Disorder (e.g., suddenly feels really scared for no reason) and Obsessive Compulsive Disorder (e.g., has to do things over and over again). The sixth subscale is Depression (e.g., feels nothing is much fun anymore). Three total scores are derived: Total Anxiety (the five anxiety subscales), Depression (one scale), and Total Anxiety and Depression (all six subscales). Raw scores are used for the analyses, but T-scores are used for interpretation in the binomial regressions to categorize whether children’s scores were in the clinical range. For reference, T-scores of 65–69 are within the borderline clinical range. T-scores of 70 + are above the clinical cut-off. Both point to a need for further clinical investigation (Chorpita et al., 2000). RCADS-P is standardized for children aged 8 years and older. Norms were derived using the most recent version (3.3) of the scoring tool.

### Statistical Analyses

A statistical analysis plan was pre-registered with AsPredicted (#13396). The pre-registered analysis plan states that linear, stepwise and binomial logistic regression models would be used. However, stepwise regressions were replaced with simultaneous regression methods because stepwise methods are considered exploratory and are best avoided outside of exploratory model building (Field, 2009). Our original plan was to explore the relationship between different subgroups within the sample (e.g., those with and without ADHD or ASD, and boys and girls). On re-consideration the decision was made prior to the commencement of analysis to remove these subgroup comparisons for three reasons. First, it would be paradoxical to compare children with and without different diagnoses such as ADHD in a study that explicitly seeks to put aside diagnostic labels to explore use of the SDQ in a transdiagnostic sample. Second, previous studies have explored the use of the SDQ in children with specific diagnoses (e.g., Simonoff et al., 2012; Bekker et al., 2016). And finally, the sample consisted of substantially more boys than girls, meaning our power to explore patterns of relationships in the girls only would have been limited. In addition to the analyses outlined in the pre-registration, confirmatory factor analyses (CFA) were run on the SDQ and RCADS to check they fit the data collected here.

The analyses conducted were as follows. Regression analyses were conducted using simple and simultaneous multiple regressions, and binomial logistic regressions. Regression analyses were followed-up with Receiver Operator Characteristics (ROC) to determine optimal SDQ cut-off scores for distinguishing children with clinical levels of Total Anxiety, Depression, and Total Anxiety and Depression. Simple and simultaneous linear regressions were used to test whether SDQ scores (total and subscales) predicted ratings of Total Anxiety, Depression, and Total Anxiety and Depression as reported in the RCADS-P. For significant predictors, a series of regression models were compared to examine the relative contribution of each significant SDQ subscale in predicting RCADS-P scores. The contributions of the different predictors were compared using change in *R*^2^ and F. The models were compared in pairs (model 1 vs. model 2, and model 2 vs. model 3 etc.) to test whether there was a significant change with the addition of each predictor. Binomial logistic regressions were then used to test whether SDQ scores (total and subscales) predicted the likelihood of a child scoring within the clinical range on the RCADS-P. Where SDQ total and subscales significantly predicted the likelihood of a clinical RCADS-P score, ROC curves were used to identify optimum SDQ cut-off scores.

The RCADS-P manual provides age-standardized T-scores, but the SDQ does not. For this reason, raw scores were entered into the analyses for both questionnaires. To check whether controlling for age affected the outcomes, the analyses were also conducted accounting for age: regression analyses were conducted using age-controlled residual scores for each subscale and total scores of the RCADS-P and the SDQ. These were calculated by predicting raw scores from age and saving the residuals for inclusion in the analyses (see Supplementary Materials). The overall conclusions did not differ when age was taken into account. For this reason, the analyses reported here are conducted on raw scores. All outcomes controlling for age are reported in the Supplementary Materials.

## Results

Descriptive statistics for all measures are presented in Table 2. Approximately half of the sample were rated within the abnormal range on the SDQ Emotion (49%), Conduct (52%), Peer Problems (47%), and Prosocial Behavior (46%) subscales. Larger proportions were in the abnormal range for hyperactivity (64%) and overall difficulties (Total 67%). Close to a third of the sample were rated as having scores within the clinical range on the RCADS for Total Anxiety (29%) and Total Anxiety and Depression (34%), and almost half were within the clinical range for Depression (43%).

**TABLE 2:** Means (SD) and Prevalence of clinical (abnormal) range SDQ and RCADS-P scores.

**Raw scores**	***M***	***SD***	**Min**	**Max**	**% Abnormal range/clinical cutoffs**
**SDQ**					
Total	19.61	7.28	1	36	67
Emotion	4.42	2.79	0	10	49
Conduct	3.75	2.6	0	10	52
Hyperactivity	7.89	2.23	1	10	64
Peer problems	3.56	2.53	0	10	47
Prosocial behavior	6.57	2.38	0	10	46
**RCADS-P (T scores)**					
Total Anxiety	31.86 (59.88)	17.66 (13.86)	0 (31)	87 (81)	29
Depression	8.98 (65.02)	5.09 (12.91)	0 (37)	23 (81)	43
Total Anxiety and Depression	40.84 (61.8)	21.54 (13.73)	1 (31)	110 (81)	34

*N = 383 (excluding outliers). Raw scores are shown for both the SDQ and RCADS. T scores for the RCADS are shown in parentheses. N (%).*

A CFA was used to test the fit of the standard five-factor structure of the SDQ in which the 25 items load on to five factors measuring Conduct Problems, Emotion, Hyperactivity, Peer Problems and Prosocial Behaviors (see Supplementary Figure 1). Fit statistics indicated that the five-factor model was an acceptable fit to the data, χ^2^(265) = 1006.44, *p* < 0.0001; RMSEA = 0.061; CFI = 0.977; SRMR = 0.074 (Schermelleh-Engel et al., 2003). Similar outcomes have been reported for participants with ADHD (Becker et al., 2006; Hall et al., 2019) and typically developing children in a similar age range to those included here (Goodman, 2001; Woerner et al., 2004; Van Roy et al., 2008; Niclasen et al., 2012).

A second CFA was conducted to test the fit of the standard 6 factor structure of the RCADS, in which the 47 items load on to measures of Separation Anxiety, Social Phobia, Generalized Anxiety Disorder, Panic Disorder, Obsessive Compulsive Disorder, and Major Depressive Disorder (see Supplementary Figure 2). Fit statistics indicated that the six-factor model was an acceptable fit to the data, χ^2^(1,019) = 2618.36, *p* < 0.0001; RMSEA = 0.071; CFI = 0.976; SRMR = 0.088. This underscores the validity of the RCADS with the current sample, and the use of the sum scales for anxiety and depression in subsequent analyses.

Significant associations were found between all subscales of the SDQ and RCADS-P (see Supplementary Figure 3). SDQ Total scores were strongly related to ratings of Total Anxiety (*r* = 0.57), Depression (*r* = 0.68), and Total Anxiety and Depression (*r* = 0.62). SDQ Emotion scores also strongly correlated with ratings of Total Anxiety (*r* = 0.75), Depression (*r* = 0.64), and Total Anxiety and Depression (*r* = 0.77), all *p* < 0.01.

### Do SDQ Total and Subscale Scores Predict RCADS Ratings of Anxiety and Depression?

Simple and multiple linear regressions were conducted to test whether SDQ scores predicted ratings of mental health. Regressions with the addition of each significant predictor were then compared to determine how much unique variance each predictor contributed to the model. Significant changes in F and *R*^2^ were recorded. Results for the simple linear and multiple regressions are summarized in Table 3, and model comparison showing significant changes in F and *R*^2^ are summarized in Table 4.

**TABLE 3:** Linear regressions predicting RCADS-P scores from SDQ scores.

	**Total Anxiety**				**Depression**				**Total Anxiety and Depression**			
**SDQ subscale**	**B**	***SE***	***B***	***p***	**B**	***SE***	***B***	***P***	**B**	***SE***	***B***	***p***
**Simple regression**												
Total	1.37	0.10	0.57	<0.001***	0.48	0.03	0.68	<0.001***	1.84	0.12	0.62	<0.001***
R^2^	0.29				0.46				0.39			
**Multiple regression**												
Emotion	4.68	0.24	0.74	<0.001***	0.92	0.07	0.50	<0.001***	5.60	0.28	0.73	<0.001***
Conduct	−0.17	0.30	−0.03	0.57	0.18	0.09	0.09	0.048*	0.01	0.35	0.001	0.98
Hyperactivity	0.70	0.30	0.09	0.02*	0.41	0.09	0.18	<0.001***	1.10	0.35	0.11	0.002**
Peer problems	0.36	0.28	0.05	0.19	0.25	0.09	0.12	0.004**	0.61	0.33	0.07	0.06
Prosocial	0.44	0.31	0.06	0.16	−0.19	0.10	−0.09	0.047*	0.25	0.37	0.03	0.50
R^2^	0.57				0.52				0.61			

*N* = 383, *p < 0.05, **p < 0.01, ***p < *0.001.*

**TABLE 4:** Model comparisons for SDQ subscales identified as significant predictors of RCADS-P scores in simultaneous linear regressions.

**RCADS-P measure**	**Model**	**SDQ measure**	**B**	***p***	***F***	**Adjusted *R*^2^**	**Difference**
Total Anxiety	A1	Emotion	0.75	<0.001***	503.18	0.569	
	A2	Emotion	0.74	<0.001***			
		Hyperactivity	0.07	0.05	255.41	0.573	Δ*R^2^* = 0.004
							Δ*F* = 3.86
Depression	B1	Emotion	0.64	<0.001***	266.35	0.411	
	B2	Emotion	0.59	<0.001***			
		Hyperactivity	0.27	<0.001***	177.22	0.483	Δ*R^2^* = 0.071**
							Δ*F* = 52.25**
	B3	Emotion	0.53	<0.001***			
		Hyperactivity	0.24	<0.001***			
		Peer problems	0.17	0.004**	129.86	0.507	Δ*R^2^* = 0.024**
							Δ*F* = 18.66**
	B4	Emotion	0.52	<0.001***			
		Hyperactivity	0.20	<0.001***			
		Peer problems	0.13	0.002**			
		Prosocial	−0.12	0.004*	101.44	0.518	Δ*R^2^* = 0.011**
							Δ*F* = 8.48**
	B5	Emotion	0.50	<0.001***			
		Hyperactivity	0.18	<0.001***			
		Peer problems	0.12	0.004**			
		Prosocial	−0.09	0.047*			
		Conduct	0.09	0.048*	82.56	0.523	Δ*R^2^* = 0.005*
Total Anxiety and Depression							Δ*F* = 3.93*
	C1	Emotion	0.77	<0.001***	555.87	0.593	
	C2	Emotion	0.75	<0.001***			
		Hyperactivity	0.12	<0.001***	293.4	0.607	Δ*R^2^* = 0.014**
							Δ*F* = 13.18 **

N = 383. *p <0.05, **p <0.01, ***p <0.001.

Simple linear regression showed that Total SDQ ratings significantly predicted Total Anxiety scores, *F*(1, 381) = 179.2, *p* <0.001, and accounted for approximately 30% of the variance. A multiple linear regression model with all subscales was significant, *F*(5, 377) = 103.2, *p* <0.0001. However, only the Emotion and Hyperactivity subscales significantly predicted anxiety ratings, and accounted for ∼57% variance (see Table 3). The model including both the Emotion and Hyperactivity (model A2) subscales accounted for marginally more variance than the Emotion subscale alone, Δ*F*(1, 380) = 3.86, *p* = 0.05, Δ*R*^2^ = 0.004 (model A1) (see Table 4). This reveals that the Hyperactivity subscale makes an important contribution to Total Anxiety scores, but it should be noted that this contribution is less than 1% after variance accounted for by the Emotion subscale is taken into account.

Total SDQ scores accounted for 46% of variance in depression ratings, *F*(1, 381) = 330.3, *p* <0.001). All five SDQ subscales, Emotion, Conduct, Hyperactivity, Peer Problems and Prosocial Behavior predicted 52% of Depression scores, *F*(5, 377) = 82.56, *p* <0.0001 (see Table 3). Each scale contributed a significant unique amount of variance and improved the model fit, all Δ*F*, *p* <0.05 (Table 4). The model comparisons revealed the best model was one including all predictors (model B5). The Emotion subscale accounted for 41% variance in depression ratings, while Hyperactivity, Δ*R*^2^ = 0.071, Peer Problems, Δ*R*^2^ = 0.024, Prosocial Behavior, Δ*R*^2^ = 0.011, and Conduct, Δ*R*^2^ = 0.005, collectively contributed a further 11% of variance.

Total SDQ scores predicted 39% of variance in combined Total Anxiety and Depression scores, *F*(1, 381) = 244.1, *p* <0.001. Multiple linear regressions revealed that both the Emotion and Hyperactivity subscales significantly predicted Total Anxiety and Depression scores, accounting for 61% of variance, *F*(5, 377) = 118.3, *p* <0.001 (see Table 3). A model including both predictors (C2) was significantly better than a model with only Emotion (C1) entered; Δ*F*(1, 380) = 13.18, *p* <0.001, Δ*R*^2^ = 0.014 (see Table 4). The Emotion subscale accounted for 59% variance in ratings, while Hyperactivity contributed a further 2% of variance.

### Do SDQ Total and Subscale Scores Predict Clinical Levels of Anxiety and Depression?

Binomial logistic regressions were used to test whether SDQ scores predicted the likelihood of a child scoring within the clinical range on the RCADS-P measures (see Table 5). Higher SDQ Total scores were significantly associated with an increased likelihood of having a score in the clinical range on all three RCADS-P scales, Total Anxiety, χ^2^(1) = 102.57, *p* <0.0001, Depression, χ^2^(1) = 144.23, *p* <0.0001, Total Anxiety and Depression, χ^2^(1) = 108.62, *p* <0.0001.

**TABLE 5:** Logistic regressions predicting clinical RCADS-P scores from SDQ scores.

	**Total Anxiety**				**Depression**				**Total Anxiety and Depression**			
**SDQ measure**	**B**	**Wald χ^2^**	***p***	**OR**	**B**	**Wald χ^2^**	***p***	**OR**	**B**	**Wald χ^2^**	***p***	**OR**
**SDQ total score**												
Total	0.19	8.41	<0.001***	1.21	0.22	9.48	<0.001***	1.25	0.19	8.67	<0.001***	1.21
Nagelkerke’s *R*^2^	0.34				0.42				0.34			
**SDQ subscale scores**												
Emotion	0.66	8.51	<0.001***	1.94	0.46	7.67	<0.001***	1.59	0.66	8.84	<0.001***	1.94
Conduct	0.01	0.15	0.88	1.01	0.15	2.22	0.03*	1.16	−0.03	−0.45	0.65	0.97
Hyperactivity	0.24	2.78	0.01*	1.27	0.22	2.96	0.003**	1.25	0.18	2.22	0.03*	1.20
Peer problems	0.01	0.08	0.94	1.01	0.02	0.31	0.76	1.02	0.06	0.95	0.34	1.06
Prosocial	0.01	0.16	0.87	1.01	−0.12	−1.77	0.08	0.89	−0.003	−0.04	0.97	1.00
Nagelkerke’s *R*^2^	0.50				0.48				0.52			

N = 383. *p <0.05, **p <0.01, ***p <0.001.

Logistic regressions including all five SDQ subscales revealed that together the subscales were significantly associated with an increased likelihood of scoring within the clinical range on Total Anxiety, χ^2^(5) = 165.81, *p* <0.0001, Depression, χ^2^(5) = 171.11, *p* <0.0001, and Total Anxiety and Depression, χ^2^(5) = 179.62, *p* <0.0001. The Emotion and Hyperactivity subscales were the only significant predictors in each case. An increase in 1 point on the Emotion subscale increased the odds of clinical range Total Anxiety scores by 1.94, Depression by 1.59, and Total Anxiety and Depression by 1.94. Similarly, an increase in 1 point on the Hyperactivity subscale increased the odds of clinical range Total Anxiety scores by 1.27, Depression by 1.25, and Total Anxiety and Depression by 1.20.

### What SDQ Cut-off Scores Predict Anxiety and Depression?

ROC analyses were used to identify optimum cut-off scores for the SDQ Emotion and Hyperactivity subscales that would indicate a child has elevated symptoms of anxiety and depression. Plotting SDQ Emotion against RCADS-P Total Anxiety and Total Anxiety and Depression showed excellent levels of discrimination (Hosmer et al., 2013): Total Anxiety, 0.900, 95% CI = 0.860–0.940; Total Anxiety and Depression, 0.901, 95% CI = 0.864–0.938). Visual inspection of the coordinates of the curve suggest an optimal cut-off score of 5 on the SDQ Emotion subscale predicts clinical levels of Total Anxiety and Total Anxiety and Depression. The SDQ Emotion and RCADS-P Depression scales showed an adequate level of discrimination (0.850, 95% CI = 0.809–0.890), and visual inspection of the curve also suggested a cut-off score of 5 on the Emotion scale predicted clinical depression scores.

Plotting SDQ Hyperactivity against Total Anxiety, Depression, and Total Anxiety and Depression showed acceptable levels of discrimination: Anxiety, 0.744, 95% CI = 0.686–0.802; Depression, 0.763, 95% CI = 0.713–0.812, Total Anxiety and Depression, 0.710, 95% CI = 0.653–0.767). Visual inspection of the coordinates of the curve suggest an optimal cut-off score of 10 on the SDQ Hyperactivity subscale predicts clinical levels of Total Anxiety, Depression and Total Anxiety and Depression.

## Discussion

The primary aim of this study was to test whether a short parent rating scale, the Strengths and Difficulties Questionnaire (SDQ; Goodman, 1997), was associated with high scores on the RCADS (Chorpita et al., 2000) measures of anxiety and/or depression in a large mixed sample of struggling learners. The SDQ has been used widely with groups of children with developmental problems categorized according to traditional diagnostic nosology (e.g., those with ADHD or ASD). This study is the first test of its utility with a heterogeneous, transdiagnostic sample of poor learners. It is both timely and important. The frequency of depression and anxiety in children and adolescents is growing (Sadler et al., 2018), and mental health difficulties are far more common in children with developmental difficulties than typically developing children (Boetsch et al., 1996; Beitchman et al., 2001; Dekker et al., 2002; Carroll et al., 2005; Morgan et al., 2008; Goh et al., 2013; McIntosh et al., 2013). Developmental problems are increasingly being studied using transdiagnostic approaches that aim to understand the processes and causes of symptoms that occur across individuals irrespective of diagnostic status (Casey et al., 2014; Sonuga-Barke and Coghill, 2014; Sonuga-Barke et al., 2016; Kotov et al., 2017). It is therefore vital to evaluate tools to identify mental health symptoms in children who are struggling at school, irrespective of diagnoses, so assessments remain useful as the field progresses.

Total SDQ scores were strong predictors of concurrent mental health problems in the current sample, as they are both for community samples (Goodman and Goodman, 2009) and children with traditionally categorized developmental disorders such as ADHD and dyslexia (Terras et al., 2009; Simonoff et al., 2012; Bekker et al., 2016). This demonstrates the validity of the SDQ as a tool with which to detect existing mental health problems in mixed groups of struggling learners, an important finding given both the move toward transdiagnostic approaches and increasing pressure on schools to identify children struggling with mental health problems (Department of Health and Education, 2018). The SDQ is freely available and simple to administer. It contains only 25 items, can be completed by parents, teachers or children themselves, and requires no clinical interpretation. The current data were collected from parents, but the items are identical for teachers and ratings between teachers and parents are often consistent (Stone et al., 2010). This suggests the tool could be used in schools with children who are falling behind cognitively and academically to determine whether they have elevated levels of anxiety and depression that would warrant further investigation.

The Emotion subscale, which includes items related to children’s mood and feelings, predicted more variance in RCADS Total Anxiety, Depression and Total Anxiety and Depression scores than Total SDQ scores. This is not surprising due to the overlap in items between the Emotion subscale of the SDQ and the RCADS. What is important, however, is that the Emotion subscale of the SDQ is shorter (only five items) and easier for parents and teachers to access and administer than RCADS and other scales that directly assess symptoms of anxiety and depression. ROC analyses identified that a cut-off score of 5 or above on the Emotion scale identified elevated levels of depression, anxiety and combined anxiety and depression on the RCADS. This score aligns with that set by Goodman (1997) in the original UK community study, and suggests the Emotion subscale cut-off is appropriate for both typically developing children and struggling learners in the United Kingdom. This is important because standard SDQ cut-off values are not valid across all samples and countries. A 2018 scoping review of studies in African nations determined that the UK-based SDQ cut-off scores were not appropriate (Hoosen et al., 2018), and in other countries, including China (Du et al., 2008) and India (Bhola et al., 2016), revised cut-off scores have been created. Revised values have also been identified for groups of children with specific neurodevelopmental differences. For example, Bekker and colleagues identified a higher Emotion subscale cut-off of 6 for determining externalizing comorbidities for a sample of children with ADHD (Bekker et al., 2016). The current data therefore reinforce the use of the Emotion subscale as a tool with which to identify concurrent symptoms of mental health problems in children who are struggling at school.

Anxiety was uniquely linked with hyperactivity in our sample of struggling learners. Although the effects were small, models containing both the Emotion and Hyperactivity subscales of the SDQ explained significantly more variance in both RCADS scales measuring anxiety (Total Anxiety and Total Anxiety and Depression) than models containing Emotion alone. Children with higher scores on Hyperactivity were also more likely to have clinical range Total Anxiety scores. Why might hyperactivity and anxious traits co-occur in this sample of poor learners? The cohort is characterized by poor executive function skills (see Holmes et al., 2020), like many other more specific groups of children with academic and learning-related problems (e.g., Carretti et al., 2009; Szucs et al., 2013; Yeniad et al., 2013). Executive function impairments are also implicated in both hyperactivity (Tripp and Alsop, 2001; Willcutt et al., 2005; Castellanos et al., 2006) and anxiety (Russell, 2003; Gotlib and Joorman, 2010; Crocker et al., 2013; Sharp et al., 2015). Executive function deficits might therefore contribute to all three characteristics of our sample: hyperactivity, anxiety and poor school achievement. A related possibility is that anxiety and hyperactivity have a shared neurocognitive basis (Schatz and Rostain, 2006). For example, executive dysfunction (Barkley, 1997; Brown, 2000; Holmes et al., 2014), dysregulated arousal (Aston-Jones et al., 2000), and abnormal patterns of brain activity and connectivity originating from the prefrontal cortex (PFC) are shared in both anxiety and hyperactivity (Levy, 2004). PFC activity is related to executive functions and gates emotional activity through projections to limbic regions such as the amygdala and nucleus accumbens. Inefficient PFC responsivity might therefore lead to both dysregulated cognitive control and emotional over-reactivity, explaining comorbid symptoms of anxiety and hyperactivity (Beesdo et al., 2009).

In contrast to anxiety, elevated symptoms of depression were linked more broadly to externalizing symptoms measured by the SDQ. Ratings on the Emotion subscale were the strongest predictor of Total Depression scores, but the other SDQ subscales also predicted a small yet significant amount of variance in depressive symptoms. The association between conduct problems and depression might reflect the presence of oppositional behaviors that can appear in childhood alongside bipolar disorder (Axelson, 2013) and form part of Disruptive Mood Dysregulation Disorder (DSM-5, 2013). For hyperactivity, depressive traits could be misdiagnosed signs of demoralization that hyperactive children experience in the face of social and school failure (Biederman et al., 1991). However, it may be that hyperactivity and depression have independent courses that can co-occur, and that the impacts of hyperactivity and interpersonal difficulties on the persistence of depression are separable but potentially additive (Biederman et al., 1998). Longitudinal data are needed to test the causal pathways between these externalizing problems and depression.

Low levels of prosocial behavior and elevated problems with peers also predicted elevated levels of depression in the struggling learners. Again, these effects were small so should not be over-interpreted, but they are consistent with a broader literature showing that children who fall behind academically are at increased risk of being bullied (Mishna, 2003; Twyman et al., 2010). Children who are bullied experience high levels of depression (Seals and Young, 2003; Brunstein Klomek et al., 2007; Perren et al., 2010) and longitudinal studies show that the relationship between bullying and depression is mediated by the quality of friendships (van Harmelen et al., 2016), prosociality (Martin and Huebner, 2007; Andrade and Tannock, 2014) and peer support (Griese and Buhs, 2014). Together with our current findings, these data suggest that interventions focussing on social experiences and skills may help build resilience against low mood in children who are struggling at school.

## Conclusion

In conclusion, the current study demonstrates that the parent version of the SDQ, and in particular the Emotion subscale, can be used to predict concurrent symptoms of anxiety and depression in a mixed sample of children who are struggling at school. At a practical level, this provides evidence for the use of the Emotion scale of the SDQ by teachers and parents to index children who may currently have elevated symptoms of anxiety and depression, removing the need to use tools such as the RCADS that are restricted to clinical use. A specific link was found between hyperactivity and anxiety, while a broader set of behaviors including conduct problems and the quality of social interactions were associated with depression alongside hyperactivity. These associations were significant, but the coefficients and effect sizes small, so caution should be exercised when interpreting them: the Hyperactivity subscale of the SDQ should not be used to index possible current symptoms of anxiety based on the current data, nor should the Hyperactivity, Prosocial or Peer Problems scales be used to detect current levels of low mood. The weak associations between hyperactivity and anxiety and depression likely reflect the well-documented comorbidity between disorders of impulsivity/hyperactivity and anxious-depressive traits (Mennin et al., 2000; Schatz and Rostain, 2006): an estimated 20–30% of children and adolescents with hyperactive disorders have a comorbid internalizing disorder (Biederman et al., 1996, 2005; Ford et al., 2003; Chronis-Tuscano et al., 2010; Rucklidge, 2010). The links between social problems and low mood point towards promoting good friendship groups and positive behaviour to affect low mood in children who struggle at school, but future longitudinal studies are needed to test the robustness of the associations reported here.

## Data Availability Statement

The data analyzed in this study is subject to the following licenses/restrictions. The data from the larger study from which these data were drawn will be made available via managed open access when the study is complete. Requests to access these datasets should be directed to calm@mrc-cbu.cam.ac.uk.

## Ethics Statement

The studies involving human participants were reviewed and approved by local NHS Research Ethics Committee (13/EE/0157). Written informed consent to participate in this study was provided by the participants’ legal guardian/next of kin.

## Author Contributions

AB and JH designed the study. The CALM Team collected the data. AB and JG completed the data analyses. AB, JG, and JH wrote the manuscript. All authors contributed to the article and approved the submitted version.

## Conflict of Interest

The authors declare that the research was conducted in the absence of any commercial or financial relationships that could be construed as a potential conflict of interest.
