# Supplementary material for: The Strengths and Difficulties Questionnaire Predicts Concurrent Mental Health Difficulties in a Transdiagnostic Sample of Struggling Learners
Source: Front Psychol. 2020 Nov 12;11:587821. doi: 10.3389/fpsyg.2020.587821 (PMC7717974; doi:10.3389/fpsyg.2020.587821)
Supplement: Supplementary file 1 [file Data_Sheet_1.docx]

Supplementary Material

# Supplementary Figures and Tables

## Supplementary Figures


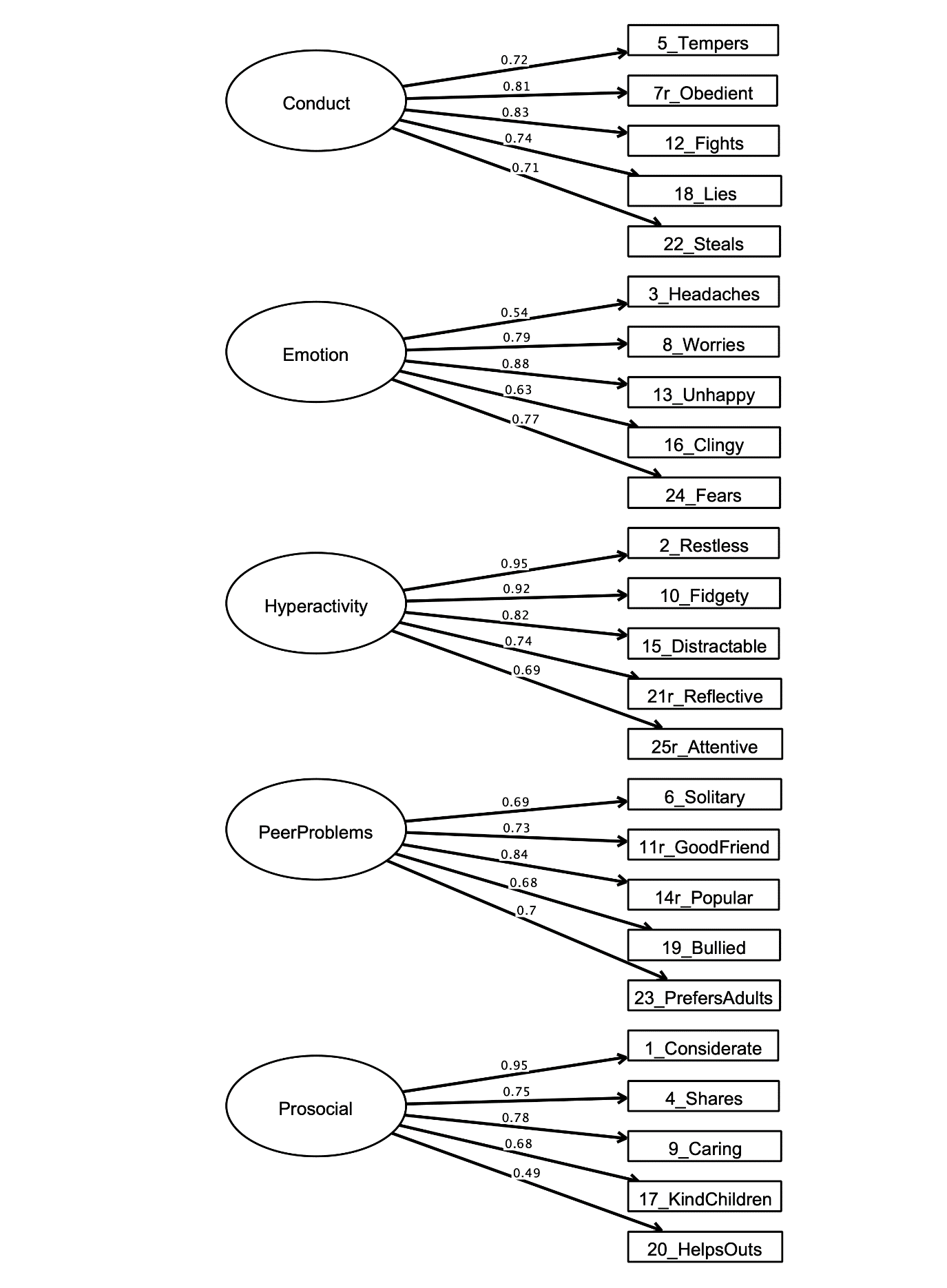
**Supplementary Figure 1**. Five factor structure of the SDQ. Latent variables representing Conduct Problems (Conduct), Emotional Symptoms (Emotion), Hyperactivity-Inattention (Hyperactivity), Peer Problems and Pro-Social Behaviour (ProSocial) are represented in ovals. Specific items are shown as observed variables in squares with item numbers and a brief description. Items 7, 11, 14, 21, 25 were reverse coded for ease of interpretation. Parameter estimates are fully standardized. Factor covariances are omitted for simplicity.


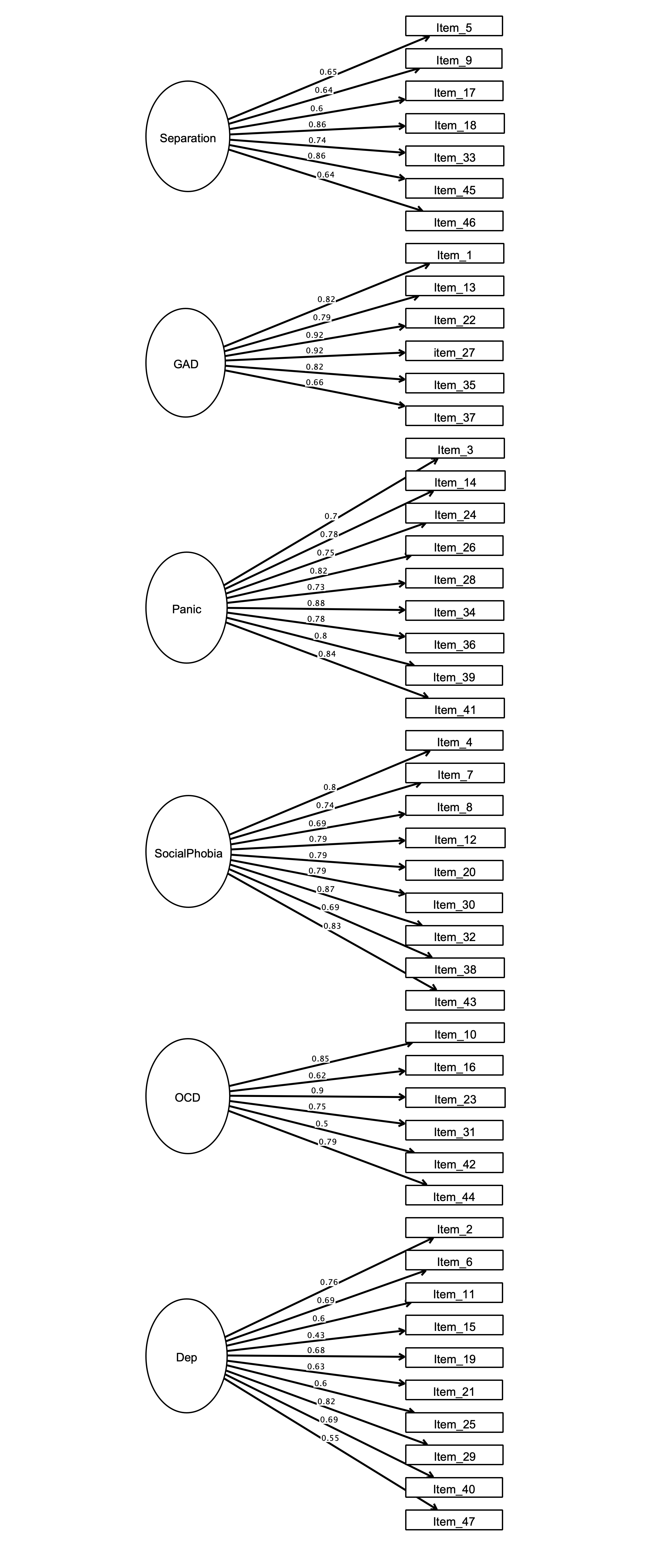


**Supplementary Figure 2**. Six factor structure of the RCADS. Latent variables representing Separation Anxiety (Separation), Generalized Anxiety Disorder (GAD), Panic Disorder (Panic), Social Phobia (SocialPhobia), Obsessive Compulsive Disorder (OCD) and Depression (Dep) are represented in ovals. Specific items are shown as observed variables in squares with item numbers. Parameter estimates are fully standardized. Factor covariances are omitted for simplicity.


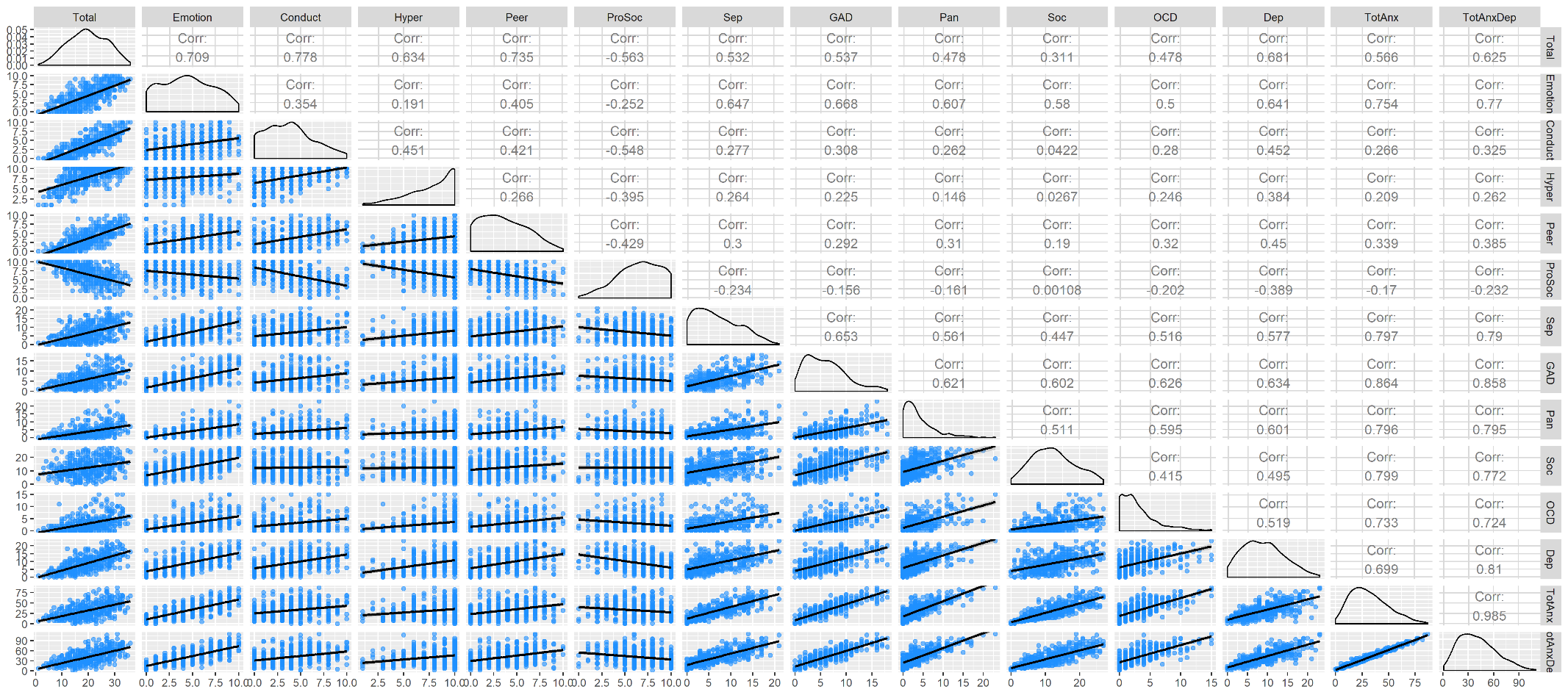
**Supplementary Figure 3.** Correlations between scores on SDQ and RCADS-P scales. All correlations were significant, *p* <.01, uncorrected. Peer = SDQ Peer Problems; ProSoc = SDQ Prosocial; Sep = RCADS- P Separation Anxiety ; GAD = RCADS- P Generalized Anxiety Disorder; Pan = RCADS- P Panic Disorder; Soc = RCADS- P Social Anxiety; OCD = RCADS- P Obsessive Compulsive Disorder; Dep = RCADS- P Depression; TotAnx = RCADS- P Total Anxiety; TotAnxDep = RCADS- P Total Anxiety and Depression.

## Supplementary Tables

**Supplementary Table 1.** Linear regressions predicting RCADS-P residual scores from SDQ residual scores

|  | *Total Anxiety* | | | | *Depression* | | | | *Total Anxiety & Depression* | | | |
| --- | --- | --- | --- | --- | --- | --- | --- | --- | --- | --- | --- | --- |
| *SDQ Subscale* | B | *SE* | *B* | *p* | B | *SE* | *B* | *p* | B | *SE* | *B* | *p* |
| *Total* | 1.37 | 0.10 | 0.56 | <.0001*** | 0.47 | 0.03 | 0.68 | <.0001*** | 1.84 | 0.12 | 0.62 | <.0001*** |
| *R²* | 0.32 |  |  |  | 0.47 |  |  |  | 0.39 |  |  |  |
| *Emotion* | 4.68 | 0.24 | 0.74 | <.0001*** | 0.91 | 0.07 | 0.51 | <.0001*** | 5.59 | 0.28 | 0.73 | <.0001*** |
| *Conduct* | -0.17 | 0.30 | -0.03 | .56 | 0.20 | 0.09 | 0.10 | .02* | 0.03 | 0.35 | 0.003 | 0.94 |
| *Hyperactivity* | 0.69 | 0.30 | 0.09 | .02* | 0.44 | 0.09 | 0.20 | <.0001*** | 1.13 | 0.36 | 0.12 | 0.002** |
| *Peer problems* | 0.38 | 0.28 | 0.05 | .18 | 0.18 | 0.08 | 0.09 | .04* | 0.56 | 0.33 | 0.06 | 0.09 |
| *Prosocial* | -0.44 | 0.31 | -0.06 | .16 | 0.18 | 0.09 | 0.09 | .05 | -0.26 | 0.37 | -0.03 | 0.48 |
| *R²* | 0.58 |  |  |  | 0.53 |  |  |  |  |  |  |  |
| *Note:* N=389. **p<.05, **p<.01, ***p<.001* | | | | |  |  |  |  |  |  |  |  |

**Supplementary Table 2.** Model comparisons for SDQ subscales identified as significant predictors of RCADS-P residual scores in simultaneous linear regressions

| RCADS-P measure | Model | SDQ measure | B | *p* | *F* | Adjusted *R*² | Difference |
| --- | --- | --- | --- | --- | --- | --- | --- |
| Total Anxiety | A1 | Emotion | 0.74 | <.001*** | 502 | .569 |  |
|  | A2 | Hyperactivity | 0.07 | .05 | 254.73 | .573 | Δ*R^2^*  = .004 |
|  |  |  |  |  |  |  | Δ*F* = 3.79 |
| Depression | B1 | Emotion | 0.64 | <.001*** | 268.62 | .41 |  |
|  | B2 | Emotion | 0.59 | <.001*** |  |  |  |
|  |  | Hyperactivity | 0.29 | <.001*** | 185.97 | .49 | Δ*R^2^*  = .081** |
|  |  |  |  |  |  |  | Δ*F* = 61.00*** |
|  | B3 | Emotion | 0.54 | <.001*** |  |  |  |
|  |  | Hyperactivity | 0.22 | <.001*** |  |  |  |
|  |  | Conduct | 0.17 | <.001*** | 134.23 | .51 | Δ*R^2^*  = .021** |
|  |  |  |  |  |  |  | Δ*F* = 16.04*** |
|  | B4 | Emotion | 0.51 | <.001*** |  |  |  |
|  |  | Hyperactivity | 0.21 | <.001*** |  |  |  |
|  |  | Conduct | 0.14 | .002* |  |  |  |
|  |  | Peer problems | 0.11 | .01* | 103.89 | .52 | Δ*R^2^*  = .009** |
|  |  |  |  |  |  |  | Δ*F* = 6.75** |
|  | B5 | Emotion | 0.51 | <.001*** |  |  |  |
|  |  | Hyperactivity | 0.20 | <.001*** |  |  |  |
|  |  | Conduct | 0.11 | .03* |  |  |  |
|  |  | Peer problems | 0.09 | .04* |  |  |  |
|  |  | Prosocial | 0.09 | .05 | 84.52 | .52 | Δ*R^2^*  = .005* |
|  |  |  |  |  |  |  | Δ*F* = 3.88* |
| Total Anxiety & Depression | C1 | Emotion | 0.77 | <.001*** | 551.78 | .59 |  |
|  | C2 | Emotion | 0.75 | <.001*** |  |  |  |
|  |  | Hyperactivity | 0.12 | <.001*** | 292.05 | .60 | Δ*R^2^*  = .014** |
|  |  |  |  |  |  |  | Δ*F* = 13.78*** |
| *Note: N*=389. **p*<.05, ***p*<.01, ****p*<.001 | | |  |  |  |  |  |

**Supplementary Table 3.** Logistic regressions predicting clinical RCADS scores from SDQ residual scores

|  | Total Anxiety | | | | Depression | | | | Total Anxiety & Depression | | | |
| --- | --- | --- | --- | --- | --- | --- | --- | --- | --- | --- | --- | --- |
| SDQ measure | B | Wald χ² | *p* | OR | B | Wald χ² | *p* | OR | B | Wald χ² | *p* | OR |
| Total | 0.19 | 8.36 | <.0001*** | 1.21 | 0.21 | 9.39 | <.0001*** | 1.24 | 0.18 | 8.56 | <.0001*** | 1.20 |
| Nagelkerke's *R*² | 0.33 |  |  |  | 0.41 |  |  |  | 0.33 |  |  |  |
| Emotion | 0.66 | 8.49 | <.0001*** | 1.93 | 0.46 | 7.64 | <.0001*** | 1.58 | 0.65 | 8.82 | <.0001*** | 1.92 |
| Conduct | 0.02 | 0.22 | .824 | 1.02 | 0.16 | 2.4 | .02* | 1.17 | -0.02 | -0.29 | .770 | 0.98 |
| Hyperactivity | 0.25 | 2.83 | .005** | 1.28 | 0.24 | 3.19 | .001** | 1.27 | 0.19 | 2.39 | .017* | 1.21 |
| Peer problems | -0.01 | -0.18 | .860 | 0.99 | -0.02 | -0.35 | .72 | 0.98 | 0.03 | 0.41 | .679 | 1.03 |
| Prosocial | -0.02 | -0.22 | .828 | 0.98 | 0.11 | 1.72 | .09 | 1.12 | -0.003 | -0.04 | .965 | 1.00 |
| Nagelkerke's *R*² | 0.50 |  |  |  | 0.47 |  |  |  | 0.51 |  |  |  |

*Note: N*=389. **p*<.05, ***p*<.01, ****p*<.001
